# Patient Input to Inform the Development of Central Nervous System Outcome Measures in Myotonic Dystrophy

**DOI:** 10.1007/s43441-020-00117-3

**Published:** 2020-01-22

**Authors:** Molly White

**Affiliations:** Myotonic Dystrophy Foundation, 1004A O’Reilly Avenue, San Francisco, CA USA 94129

**Keywords:** myotonic dystrophy, PFDD, CNS, endpoint

## Abstract

Myotonic dystrophy type 1 (DM1) and myotonic dystrophy type 2 (DM2) are multisystem, genetic disorders caused by repeat expansions on chromosome 19 (DM1) and chromosome 3 (DM2). Although the effects of DM on the skeletal, cardiac, and smooth muscles, as well as the endocrine and central nervous systems, can be disabling, there are no disease-modifying therapies for the disorder. Following a process established by the US Food and Drug Administration (FDA) in 2012 known as the Patient-Focused Drug Development (PFDD) Initiative, Myotonic (formerly the Myotonic Dystrophy Foundation) has been conducting patient- and caregiver-inclusive sessions to explore disease burden as defined by patients and caregivers, and what affected individuals want most from potential new therapies. In September 2017, at Myotonic’s annual conference, a session titled “Bringing the Patient Voice to CNS-Targeting Drug Development in Myotonic Dystrophy” attracted some 350 members of the DM community. During the session, patients and caregivers described CNS disease symptoms, their impact on quality of life, and potential CNS-related targets that they considered important for drug development consideration. These included fatigue and daytime sleepiness; dysregulated sleep; cognitive deficits such as “brain fog,” memory and focus impairment, learning and attention difficulties, and time management challenges; emotional/psychological/behavioral difficulties, including impulsivity, apathy, antisocial behavior, personality changes, and depression; social difficulties, including disconnection, lack of awareness, and feelings of isolation; and general anxieties about the future and potential loss of independence. Improvements in memory and lessening of “brain fog” were considered particularly important.

## Introduction

Myotonic dystrophy (DM), the most common type of adult-onset muscular dystrophy with a population-based prevalence of 1:2300 (myotonic dystrophy type 1, Johnson 2018), is a progressive genetic disorder that affects multiple systems in the body, including the central nervous system (CNS) [1]. Given the lack of FDA-approved therapies, the need is great for therapies that will slow, halt or reverse the disease, or provide meaningful relief from its debilitating symptoms.

In September 2016, Myotonic (formerly the Myotonic Dystrophy Foundation) convened a patient-focused drug development meeting to help researchers, regulators, and drug developers understand the burdens and symptoms of DM and their impacts on patients’ daily lives, as well as patients’ assessments of clinically meaningful benefit of future approaches to treating and managing DM.

This meeting followed a process established by the Food and Drug Administration’s (FDA’s) Patient-focused Drug Development (PFDD) Initiative in 2012, the purpose of which is to bring the patient voice into regulatory decision-making by helping FDA understand the clinical context of the condition more comprehensively and directly from patients and their caregivers [2].

Under the PFDD Initiative, the FDA convened 20 meetings across a range of diseases between 2013 and 2017. In 2015, it launched a parallel effort to allow disease-specific organizations like Myotonic to convene externally led meetings in which FDA officials participated. The Myotonic PFDD meeting was the first meeting of this type, and it resulted in a Voice of the Patient Report, which was published by Myotonic and submitted to the FDA in May 2017 [3].

Following the Myotonic PFDD meeting, DM researchers and others in the DM community noted the need for a more robust discussion of DM symptoms, particularly in the brain. Indeed, while muscle weakness was reported by patients and caregivers at the Myotonic PFDD meeting as the most prominent symptom of DM, many patients said that CNS-related symptoms, such as fatigue, daytime sleepiness, and cognitive dysfunction, constituted a more significant burden. However, relative to the musculoskeletal manifestations, CNS-related symptoms were not as well-characterized in terms of their impacts on patients’ daily lives.

In September 2017, at the Myotonic Annual Conference, a session called “Bringing the Patient Voice to CNS-Targeting Drug Development in Myotonic Dystrophy” attracted some 350 members of the DM community (those living with the disease and their caregivers) to share their stories. The goal of the session was to provide drug developers and regulators with information about the burdens of CNS-related symptoms, as described by DM patients and caregivers, and ultimately to help them create appropriate CNS-related outcome measures for use in clinical trials.

## Etiology of CNS Involvement in DM

There are two major types of DM: type 1 (DM1) and type 2 (DM2). DM1 and DM2 are caused by genetic expansion mutations, although the mutations occur in genes on different chromosomes. DM1 is caused by an expansion mutation on chromosome 19, and DM2 results from an expansion mutation on chromosome 3. Both types are members of a group of diseases called RNA repeat expansion diseases, where a sequence of three or four nucleotides in the DNA and RNA are replicated many times [4]. The number of replications roughly determines disease severity, but this number is unstable and increases, both over a person’s lifetime and when passed to the next generation. As a result, the severity of disease symptoms varies substantially, even among family members, and is unpredictable.

DM1- and DM2-causing mutations lead to changes in the levels of many cellular proteins, although not all the effects of these complex mutations are yet understood at the molecular level. In the brain, structural changes believed to result from DM-causing mutations can be seen in magnetic resonance imaging (MRI) scans as changes in brain structure and disruption of neuronal networks. These alterations compromise information processing and can be detected by neuropsychological tests as impairments in executive function, attention, memory, and visuo-spatial function [5]. It is thought that these impairments may contribute to many of the symptoms reported by patients, such as “brain fog,” sleepiness, and other cognitive difficulties described by patients and observed by their direct caregivers.

## Patient and Caregiver Perspectives on CNS Symptoms

The session on CNS-related symptoms in DM at the September 2017 Myotonic conference was structured to elicit from patients and caregivers their experiences living with this subset of disease symptoms as well as their perspectives on the adequacy of available therapies to treat these specific symptoms (Table 1). James Valentine, JD, introduced the session and explained the format and objectives of the meeting. A panel of 2 caregivers, one adult with DM2, one adult with adult-onset DM1, and two adults with juvenile-onset DM1 read personal remarks addressing the key questions posed to the panel (see below). Mr. Valentine then invited other meeting attendees to participate in a facilitated open discussion. Members of the DM community (6000 + individuals living with the disease and their caregivers and immediate family members who have opted into the Myotonic community) unable to attend the meeting were also encouraged to submit written statements online, via monthly newsletter outreach, social media posts, and communication from Myotonic support group facilitators. Very few responses were received online and those that were corresponded very closely with responses from participants at the conference.

**Table 1.** CNS Symptom Burden by Domain.

CNS Symptom Domain	Related Symptoms
Fatigue/excessive daytime sleepiness	∙ Hypersomnia ∙ Fatigue/tiredness ∙ Sleep apnea ∙ Narcolepsy ∙ Difficulty waking ∙ Non-restorative sleep ∙ Auditory hallucinations ∙ Sleep-related paralysis ∙ Vivid dreams
Cognition	∙ Brain fog ∙ Attention deficit ∙ Executive function deficits ∙ Memory & focus impairments ∙ Learning difficulties/delays ∙ Time management issues
Emotional/social	∙ Anxiety ∙ Depression ∙ Impulsivity ∙ Apathy ∙ Personality changes ∙ Antisocial behavior

The 300 + patient and caregiver cohort participating in this session contains some performance bias, in that they were able to travel to the Myotonic Annual Conference and manage the physical and intellectual demands of a 2-day learning and networking event. They tend to be engaged with Myotonic, receive the monthly newsletter, sometimes join virtual or onsite support groups, and follow news about the disease. That said, the overall description of CNS symptom burden aligns closely with other published studies, notably the survey-based *Christopher Project* [6], and the *Consensus*-*based Care Recommendations* published by Myotonic [for Adults with Myotonic Dystrophy Type1 [7], adults with myotonic dystrophy Type 2 [8], and patients with childhood-onset myotonic dystrophy type 1 [9], adding validity to the experiences described below.

### Discussion Questions for DM Patients and Caregivers

**Table:** 

Burden of CNS-related symptoms of DM
Of all the CNS-related symptoms that you experience because of DM, which symptoms have the most significant impact on your life?
Are there specific activities that are important to you but that you cannot do at all or as fully as you would like because of your CNS-related symptoms?
How do your CNS-related symptoms and their negative impacts affect your daily life on the best days and on the worst days?
How have your condition and its symptoms changed over time?
What worries you most about your CNS-related symptoms?
Approaches to treatment of CNS-related symptoms of DM
What are you currently doing to help treat CNS-related symptoms? How well do these things control your condition?
Assuming there is no complete cure for your CNS-related symptoms, what specific things would you look for in an ideal treatment for your condition?

Input from the patients and caregivers from this session is summarized below. The patients’ and caregivers’ own words are used as much as possible in order to represent their experiences and preferences, rather than interpret their comments.

### Experience of Living with CNS Impacts of DM

Both patients and caregivers cite CNS symptoms as significantly burdensome. In some cases, particularly patients with juvenile-onset myotonic dystrophy, CNS symptoms are the most problematic of the symptoms with which they live. Disrupted and inadequate sleep, fatigue, brain fog, and focus deficits are leading impacts cited by both patients and caregivers, as well as anxiety and depression. Importantly, patients and caregivers are aligned with respect to their understanding of the CNS symptom burden, usually describing the symptoms and impacts similarly. In some cases, patients may not perceive overall burden to be as impactful as their caregivers. However, the shared understanding of CNS symptom burden adds credibility to the patient voice in identifying and understanding CNS symptom burden in myotonic dystrophy.

Patient and caregivers reported a range of CNS symptoms, ranging from fatigue and daytime sleepiness to sleep problems and emotional, psychological, and behavioral difficulties. Session participants indicated that the burden of these CNS symptoms is high. One adult with juvenile-onset DM1 said, “I feel that my CNS issues are a primary concern.” A caregiver added, “The physical symptoms are devastating in and of themselves. The symptoms in the brain run the gamut of simply annoying to potentially fatal.” (https://www.myotonic.org/living-dm-patients-report-changes-over-time-2018-mdf-annual-conference).

While many CNS symptoms experienced by patients were described as being DM-related, the nature of these manifestations made it difficult for patients and their loved ones to know whether or not symptoms were a result of DM. For example, caregivers expressed frustration at not understanding how the CNS manifestations of the disease affect their loved ones.

One man who cares for his wife living with adult-onset DM1 and 5-year-old son with congenital DM1 said, “She’ll tell me she’s tired. … I know a lot of it is the disease but I don’t understand what she’s going through. … Sometimes she is selfish. Is that her personality or part of the disease?” He went on to say, “Nothing has been done to address what we can do [on] our side to help out. I have to remind my 10-year-old that it’s not Mom’s fault; it’s the disease. I need more tools in my toolbox to help me communicate and get through the fog” (https://www.myotonic.org/living-dm-patients-report-changes-over-time-2018-mdf-annual-conference).

As shown in the following remarks, CNS symptoms described by patients and caregivers affect their quality of life and ability to live an independent life. What follows are quotes from participants, grouped according to common themes that emerged. All quotes and related content from patients can be accessed on the Myotonic website via this link: https://www.myotonic.org/living-dm-patients-report-changes-over-time-2018-mdf-annual-conference.

*Fatigue and daytime sleepiness* were perhaps the most common and disabling CNS symptoms reported by DM patients. Fatigue may be related, in part, to muscle weakness in DM. However, some studies suggest that CNS dysfunction, including depression and sleep-related problems, may result in mental fatigue [10]. Furthermore, patients and caregivers tend to use different wording to describe CNS symptoms and their associated burden than that used to describe physical muscle weakness or fatigue (Voice of the Patient Report: Myotonic Dystrophy Externally led Patient-Focused Drug Development Meeting, September 2016). Muscle-related symptom language frequently relates to difficulty or inability to complete particular physical functions or tasks (e.g., lifting the arms, standing). CNS symptom burden language typically refers to a general lack of energy or tiredness:

“I deal with extreme fatigue and daytime sleepiness… I would sometimes fall asleep in class.” (young adult with juvenile-onset DM1)

“I have fallen asleep while working on my computer, while doing paperwork at my desk, during top level management meetings; the list goes on and on.” (adult with DM2)

“I’m so tired I just can’t do anything… It’s almost like you feel sick, your eyes are really heavy… I can’t even move my body.” (adult with DM1)

“As a child I was distracted and would tire easily. Now I nap for four hours or more but am always tired.” (young adult with juvenile-onset DM1)

“My son says the worst daily thing is the extreme sleepiness he feels driving home after college or work.” (mother of adult son living with juvenile-onset DM1)

*Sleep problems* were also a nearly universal complaint of DM patients. These problems included difficulty waking up in the morning, disrupted sleep, narcolepsy and sleep apnea. The inability to sleep soundly may be related to fatigue, as well as to many of the other behavioral, cognitive, psychological and social difficulties that were also reported.

### Difficulty Waking Up in the Morning

“It takes me about an hour to full[y] wake up and function… I’m easily agitated and frustrated in the morning.” (adult with juvenile-onset DM1)

“I wake up feeling tired. I have never woken up feeling refreshed.” (adult with juvenile-onset DM1)

“I find it nearly impossible to wake [my son] up in the morning and get him to take his first pill.” (caregiver of adult with juvenile-onset DM1)

### Disrupted Sleep, Vivid Dreams, and Difficulty Distinguishing Between Sleep and Awake States

“At night I have trouble falling asleep and staying asleep.” (adult with adult-onset DM1)

“I have had auditory hallucinations and sleep-related paralysis just prior to falling asleep and just after waking. I have tried numerous drugs and therapies and nothing seems to help for more than a week or two at a time.” (adult with DM2)

“I dream so vividly I think I’ve done things,” said one woman who also said she does not sleep well and has sleep paralysis and narcolepsy. (adult with adult-onset DM1)

“I have dreams where you think you are awake and doing things. You don’t know what’s real.” (young adult with juvenile-onset DM1)

“Dreams are so vivid, I think they happened.” (adult with juvenile-onset DM1)

“The vivid dreams—you think you have done something you haven’t done. I thought I was crazy.” (young adult with juvenile-onset DM1)

“I’m starting to have crazy dreams. Even though things didn’t happen, it’s hard to let go of the emotions.” (adult with DM2)

After hearing the testimony of meeting participants, one woman wrote: “Our conversation tonight was about my husband, son, and daughter dreaming so vividly they believe it happened. We never discussed this or thought anything of it until hearing so many people mention vivid dreaming at the conference patient session.”

*Cognitive problems* were also widely reported, ranging from what many people called “brain fog” to specific concerns about impairments in memory, attention, executive function and learning. These patient reports support research studies suggesting wide variability in cognitive function among people with both DM1 and DM2 [11].

### Brain Fog

“Simple things like responding to yes and no questions are often followed with nonsense and non-sequiturs,” said a man who cared for his wife with DM1. “Sometimes it could take over a minute to complete a simple statement and [this caused] her a great deal of frustration and anger.”

“Brain fog is getting worse. Words on the tip of my tongue just go. [I used to be an accountant], but now I can’t pick up a form anymore and look at it—it starts spinning.” (adult with DM2)

### Memory and Focus Impairment

“When watching a movie, I usually end up rewinding scenes over and over and over again to fully grasp the story line.” (adult with juvenile-onset DM1)

“If you speak too long, I will forget what you said in the beginning. I probably will interrupt you because I have forgotten what you had been saying. … When I read, by the time I get to the end of the page, I have forgotten what I read.” (adult with juvenile-onset DM1)

“I spent 13 years giving presentations. … I could memorize, understand, present and improvise with almost zero difficulty. …This has all changed drastically over the last eight years. …The ability to memorize is pretty much gone. Improvising is out of the question; if I am not reading from a double-spaced script, I will simply forget where I am at or what I am trying to say. … Sometimes I will say the opposite of what I mean.” (adult with DM2)

A 76-year-old woman with a family history of DM1 said, “It can be frustrating. Remembering to take the right pills, getting to the bathroom before it’s an emergency, getting to meetings on time. … A few days ago, I went to lunch with some girlfriends. After lunch I suggested that we have dessert … so we left with me leading the way—right past the [cupcake restaurant] and right on down the street to the car…”

### Learning Difficulties

“It took me three or four times longer than my coworkers to learn the cash register. … I would make a mistake and then get frustrated, defensive and agitated.” (adult with juvenile-onset DM1)

“I look at a diagram now, and it is like reading a foreign language. … I just can’t process the information.” (adult with adult-onset DM1)

### Attention Difficulties

“I’m not able to drive, partly due to my attention deficit disorder.” (adult with juvenile-onset DM1)

“My attention span is very short. I think this has gotten worse since I was a teenager. I get distracted very easily, and then I don’t know what I was doing. … I lose interest before I finish almost anything.” (adult with juvenile-onset DM1)

“It is extremely difficult to hold a train of thought. … My mind will wander off. … On bad days I may have to read and reread a page four or five times in order to catch onto the concept. … Any type of stress or pressure seems to exacerbate all these issues about 10-fold.” (adult with juvenile-onset DM1)

“My son has always had the attention span of a caffeinated gnat,” said a man who cared for his wife with DM1 and son with congenital DM1. He said his wife crashed her car when she did not see a red light. “It could have been the result of her limited attention span,” he said.

“My son has difficulty concentrating, and it has worsened over time.” (caregiver of adult with juvenile-onset DM1)

### Time Management Issues

“Time management is something that impacts me a lot. I can’t wrap my head around getting somewhere on time. I show up either late or early.” (adult with juvenile-onset DM1)

Emotional, psychological and behavioral difficulties were also reported, particularly anxiety and impulsivity. In some cases, these symptoms were extremely severe, and many people described how these symptoms have worsened over time.

### Impulsivity

“I sometimes explode and get angry over the littlest things. … I know I have a hard time keeping my emotions under control, and these symptoms have become more and more frequent over the last few years.” (adult with juvenile-onset DM1)

“I lack impulse control. I tend to say things without thinking about what I have said or thinking about another person’s feelings.” (adult with juvenile-onset DM1)

“I sometimes am aggressive and impulsive and take things out on my students. These behaviors have gotten worse over time.” (adult with adult-onset DM1)

“My son had no way to determine that there would be consequences for his behavior; he would just act impulsively.” (caregiver of adult with childhood-onset DM1)

### Apathy

“My son’s apathy is his greatest challenge, along with making good choices. He even consciously doesn’t take his methylphenidate when he wants to ‘lay around all day.’” (caregiver of adult with juvenile-onset DM1)

### Antisocial behavior

“My son tested as having antisocial personality disorder. … When he was in school, he distracted all the other students, had nervous energy and talked constantly, so the teachers became very frustrated with him. … The worst thing was, he molested his sister. He was in a psychiatric hospital for that. … He went to jail. He couldn’t hold a job so he was homeless a lot…. I couldn’t allow him to live with me or I wouldn’t have survived.” (caregiver of adult with childhood-onset DM1)

### Personality Changes

“My father is 68. The rate at which his personality has changed has progressed more rapidly as his physical symptoms have worsened.” (adult with adult-onset DM1 re: her father with same diagnosis)

“Mom’s personality changed drastically; she was vicious to one of her friends.” (daughter of adult with DM2)

### Depression

A man who cared for his wife with DM1 said depression was one of the most difficult problems. (caregiver of adult with adult-onset DM1)

Social problems experienced by people with DM were often attributed to fatigue, memory impairments, impulsivity, anxiety and behavior problems. However, there were also suggestions that some people with DM may have more general difficulty connecting with others.

### Disconnection

“My interactions with family, friends and coworkers have been affected by my behaviors. I now worry that they will continue to worsen over time.” (adult with juvenile-onset DM1)

“I have many acquaintances but few friends. I forget to call people. I don’t reach out to others. … I don’t go out to eat because of my anxiety with people watching me.” (adult with juvenile-onset DM1)

“My parents remind me to ask questions to show interest in other people.” (adult with juvenile-onset DM1)

Speaking of his son who had congenital DM1: “He was often withdrawn and quiescent. … He enjoyed living his own world.”

Speaking of her daughter with juvenile-onset DM1: “She has trouble reading people. She makes friends easily but doesn’t develop healthy friendships.”

### Lack of Awareness, Naivete

Although most of the patients who spoke at the meeting were well aware of the difficulties they attribute to DM, some caregivers described family members as being “in denial” about their disease symptoms or showing a remarkable lack of awareness and judgment that resulted in social problems, some of which had serious consequences. This lack of awareness has also been observed in research studies [12].

“The individuals in my family don’t believe they have any CNS or cognitive issues,” said one woman who cares for a husband and four children with DM1. “My family members have a naivete that is almost childlike. Due to this lack of awareness and naivete, they have become vulnerable victims many times.” (caregiver of adult children with juvenile-onset DM1)

“I can give you examples of how people [with DM] have been exploited due to their poor judgement,” said the mother of three adult children with DM. “For example, [our daughter] has been with her boyfriend and his two kids for seven years.… They both have poor judgment and they always have a couple of homeless people living with them. Their mortgage doesn’t get paid and they run to us.” (caregiver of adult children with juvenile-onset DM1)

“In the last year, I have seen [my father’s transition] to anger and ability to filter during conversations change noticeabl[y] during over a period of weeks. He will tell you that he is fine and nothing is wrong. He is unaware of this change.” (caregiver of adult children with juvenile-onset DM1)

“My son met a girl when he was 22 who took advantage of his kindness and generosity. … During the four months he was not living here, she convinced him that we were terrible and disrespectful to them. The end result is that he spent $14,000 in four months, and that was every dollar he had.” (caregiver of adult children with juvenile-onset DM1)

### Feeling Isolated and Misunderstood

Meeting participants described a feeling of isolation from family, friends and coworkers. Many of the symptoms of DM, such as the debilitating fatigue and forgetfulness, are invisible and variable, making it difficult for others to appreciate their impact.

“My husband doesn’t understand. My employer doesn’t believe I’m really sick. People look at me and say, ‘You look fine.’” (adult with adult-onset DM1)

“My husband can’t accept what’s happening; he’ll say, ‘Just try harder.’” (adult with adult-onset DM1)

“I want doctors and people to understand that this is a real disease that affects all parts of our lives.” (adult with adult-onset DM1)

*Fears about the future and concerns related to loss of independence* were expressed by several participants. The progressive nature of the disease and its implications for leading an independent life elicit fear about what the future holds among both patients and caregivers. For some individuals with DM, the progression of physical and cognitive symptoms can mean they will at some point need to rely on others for support in their daily lives.

**“**A meaningful benefit of a future treatment would allow me to lead a more independent life, like driving a car, keeping a job so I could be financially independent and, most importantly, being able to care for my 5-year-old son.” (caregiver of adult children with juvenile-onset DM1)

“We need to be able to function as normal human beings. This disease is stripping away far too much from our lives to let it continue with no treatments, therapies or medications for the CNS-related problems of DM2.” (adult with DM2)

“My mom [with DM2] died in April. Now that she’s gone, I’m having symptoms and it’s starting to scare me. I haven’t been diagnosed, but I know I have it. I’m afraid to tell my doctor because of insurance.” (adult with DM2)

### Perspectives on Treatments for CNS Symptoms in DM

There are currently no treatments to impede the progression of DM. Thus, non-DM-specific treatments focus solely on relieving symptoms and preventing complications of the disease [1]. As research moves forward to identify novel therapies [13, 14], it will be essential for regulators to understand how patients’ and caregivers’ view the utility of these existing products, as well as the preferences for what they hope these new therapies will provide.

*The benefits and limitations of modafinil, a wakefulness-promoting drug*, were noted by some participants. Some said the drug was effective in reducing daytime sleepiness, but one caregiver of an adult with DM1 said the drug gave his wife energy without initiative. Modafinil is not specifically labeled for use in DM, and some patients reported difficulty gaining access to it for this reason, implying that having a drug specifically labeled for use in DM would be welcome. Other participants reported taking Ritalin and other stimulant medications to manage CNS symptoms such as sleepiness and difficulty focusing.

*Improvements in memory and lessening of “brain fog”* were mentioned as important aspects of any treatment.

“I would like to see in a therapy an improvement on memory. … A meaningful benefit of a future treatment would allow me to lead a more independent life.”

“We need something to slow these effects of DM2 on our brains, or better yet to help reverse some of these effects. It would be nice to see a medication [that would] help increase the lost cognition and decrease brain fog.” (adult with DM2)

*The importance of treatment for CNS symptoms* was expressed by some participants in statements like the following.

“I’ll try anything to slow it down or stop it.” (adult with adult-onset DM1)

“I hope, beg and pray for a drug or therapy that will help all the brain-related symptoms that DM1 patients are experiencing.”

## Conclusion

It appears that patients and caregivers prefer a drug that will slow one or more of the CNS symptoms of DM, with hopes that such a therapy will increase independence in activities of daily living and allow patients to sustain or regain their sense of self.

The deficits experienced by people with DM resemble those seen in other brain disorders, including problems with memory, attention, language, executive function, decision-making, sleep, and depression, as well as changes in behavior and personality. As with these other diseases, the heterogeneity and variability across subpopulations have complicated efforts to identify endpoints that would enable the successful development of new treatments.

In some other disease areas, such as schizophrenia, academic and industry researchers have joined with patients and regulators to identify such endpoints [15]. In Alzheimer’s disease (AD), the Alzheimer’s Disease Neuroimaging Initiative (ADNI) has been established to better understand the biology of AD and identify biomarkers [16]. Similar consortia approaches may be needed to accelerate treatment development for DM.

For any such consortium to achieve its goals and align with the needs of regulatory agencies, incorporation of the patient voice will be essential. This session on CNS symptoms of myotonic dystrophy can serve as a foundation for identifying CNS concepts of interest to study in DM clinical trials, develop CNS-related outcome assessments to measure treatment-related changes, and inform researchers about the degree of change that is meaningful to patients. As the FDA has learned through its PFDD Initiative, patients and caregivers are the disease experts and must be a part of the drug development process.

## References

[1] Thornton CA. Myotonic dystrophy. *Neurol Clin.* 2014;32(3):705–19, viii. 10.1016/j.ncl.2014.04.011 PMC4105852 25037086

[2] Perfetto EM, Burke L, Oehrlein EM, Epstein RS (2015). Patient-focused drug development: a new direction for collaboration. Med Care.

[3] Foundation MD. Voice of the Patient Report: Myotonic Dystrophy 2016.

[4] Ranum LP, Cooper TA (2006). RNA-mediated neuromuscular disorders. Annu Rev Neurosci.

[5] Baldanzi S, Cecchi P, Fabbri S, Pesaresi I, Simoncini C, Angelini C (2016). Relationship between neuropsychological impairment and grey and white matter changes in adult-onset myotonic dystrophy type 1. Neuroimage Clin..

[6] Hagerman K (2019). The myotonic dystrophy experience: a North American cross-sectional study. Muscle Nerve.

[7] Ashizawa T, Gagnon C, Groh W (2018). Consensus-based care recommendations for adults with myotonic dystrophy type 1. Neurol Clin Pract..

[8] Schoser B, Montagnese F, Bassez G (2019). Consensus-based care recommendations for adults with myotonic dystrophy type 2. Neurol. Clin Pract..

[9] Johnson N, Aldana E, Angeard N (2019). Consensus-based Care Recommendations for congenital and childhood-onset myotonic dystrophy type 1. Neurol Clin Pract..

[10] Angelini C, Tasca E (2012). Fatigue in muscular dystrophies. Neuromuscul Disord..

[11] Peric S, Rakocevic Stojanovic V, Mandic Stojmenovic G, Ilic V, Kovacevic M, Parojcic A (2017). Clusters of cognitive impairment among different phenotypes of myotonic dystrophy type 1 and type 2. Neurol Sci..

[12] Baldanzi S, Bevilacqua F, Lorio R, Volpi L, Simoncini C, Petrucci A (2016). Disease awareness in myotonic dystrophy type 1: an observational cross-sectional study. Orphanet J Rare Dis..

[13] Ho G, Cardamone M, Farrar M (2015). Congenital and childhood myotonic dystrophy: current aspects of disease and future directions. World J Clin Pediatr..

[14] Turner C, Hilton-Jones D (2014). Myotonic dystrophy: diagnosis, management and new therapies. Curr Opin Neurol.

[15] Green MF, Nuechterlein KH (2004). The MATRICS initiative: developing a consensus cognitive battery for clinical trials. Schizophr Res.

[16] Weiner MW, Veitch DP, Aisen PS, Beckett LA, Cairns NJ, Cedarbaum J (2015). 2014 Update of the Alzheimer’s Disease Neuroimaging Initiative: a review of papers published since its inception. Alzheimers Dement..

